# Supplementary material for: High Expression of HERV-K (HML-2) Might Stimulate Interferon in COVID-19 Patients
Source: Viruses. 2022 May 7;14(5):996. doi: 10.3390/v14050996 (PMC9143815; doi:10.3390/v14050996)
Supplement: Supplementary file 1 [file viruses-14-00996-s001.zip › viruses-1695747-supplementary.pdf]

Supplementary

Table S1. HERV-K(HLM-2) *gag*, *env*, *pol* gene high expression site.

	Chrom	Start	End	Span	Value
**HERV-K(HLM-2) *env***	chr3	101699823	101699990	168	1.929673961
	chr1	155627690	155627857	168	1.784949165
	chr11	118722038	118722205	168	1.784949165
	chr12	58328483	58328650	168	1.784949165
	chr1	75382267	75382434	168	1.780737525
	chr22	18946661	18946828	168	1.775836426
	chr19	76123	76288	166	1.740909801
	chr19	27638641	27638808	168	1.736138013
	chr5	156658730	156658897	168	1.72804777
	chr8	139460930	139461097	168	1.72804777
	chr6	77717961	77718128	168	1.718362357
	chr8	182002	182169	168	1.701782086
	chr8	7498899	7499066	168	1.666382388
	chr1	207636128	207636292	165	1.654442711
	chr8	720288	720455	168	1.617793389
	chr10	6825205	6825370	166	1.593812609
	chr16	34413075	34413241	167	1.53972898
	chr11	101703337	101703501	165	1.516085483
	chr16	34998589	34998755	167	1.471027744
	chr7	4591954	4592121	168	1.410600995
	chr5	30487671	30487836	166	1.361065277
	chr1	160698825	160698988	164	1.275826241
	chr2	207636128	207636292	165	1.27495668
	chr7	4583450	4583617	168	1.154509047
	chr22	1251	1395	145	1.070899471
	chr3	185563574	185563739	166	1.053087193
	chr13	28851	28995	145	0.996626025
	chr21	29396	29541	146	0.996626025
	chr21	18561649	18561817	169	0.74853494
	chr19	11478	11645	168	0.567922824
	chr3	9848703	9848870	168	0.496155398
	chr19	386477	386621	145	0.452300272
	chr3	113025295	113025460	166	0.44016123
	chr19	35572494	35572660	167	0.105466135
	chr3	125898575	125898736	162	0.10325325
	chr19	22580569	22580735	167	0.004983448
**HERV-K(HLM-2) *gag***	chr12	58335037	58335251	215	1.697430738

	chr3	185569852	185570067	216	1.547914652
	chr6	77724495	77724710	216	1.547914652
	chr7	104751911	104752126	216	1.547914652
	chr7	4590018	4590233	216	1.542251387
	chr7	4598521	4598736	216	1.542251387
	chr19	69810	70025	216	1.525179958
	chr1	155633967	155634188	222	1.068516936
	chr8	7505467	7505681	215	1.068516936
	chr3	148566699	148566913	215	1.067547548
	chr19	18047	18261	215	1.066782242
	chr8	713673	713887	215	1.063812317
	chr22	18940338	18940552	215	1.063812317
	chr3	113031565	113031786	222	1.063618977
	chr8	188570	188784	215	1.063618977
	chr19	27645543	27645765	223	1.063618977
	chr8	139462083	139462297	215	1.061884283
	chr5	156665007	156665221	215	1.038310948
	chr3	125891958	125892172	215	1.018582994
	chr4	160660283	160660497	215	1.006245185
	chr11	118728297	118728510	214	0.730855891
	chr5	30494237	30494484	248	0.11339395
	chr11	101696727	101696941	215	0.086024696
	chr21	18567930	18568144	215	0.039789321
	chr1	75378750	75378940	191	0.01808763
**HERV-K(HLM-2)** ***pol***	chr7	4586046	4586188	143	4.752136354
	chr3	185565876	185566015	140	2.378069925
	chr8	184598	184737	140	2.378069925
	chr8	717720	717859	140	2.378069925
	chr22	18944385	18944524	140	2.378069925
	chr5	156661034	156661173	140	2.374066429
	chr8	7501495	7501634	140	2.374066429
	chr19	27641238	27641375	138	2.368090493
	chr12	58331076	58331215	140	2.326021808
	chr10	99823079	99823218	140	2.311872523
	chr1	155629994	155630132	139	2.243889052
	chr10	6827797	6827936	140	2.243889052
	chr5	30490261	30490400	140	2.237567373
	chr3	113027594	113027732	139	2.233909619
	chr19	14075	14212	138	2.233909619
	chr11	101700769	101700907	139	2.233909619
	chr19	73851	73989	139	2.233909619
	chr11	118724337	118724475	139	2.209885012

	chr21	18563954	18564093	140	2.207663966
	chr13	26576	26714	139	1.966499592
	chr3	101697551	101697688	138	1.87880392
	chr3	125896006	125896143	138	1.58149078
	chr1	160696555	160696691	137	0.453238512
	chr19	37110233	37110367	135	0.386869425
	chr1	150633558	150633697	140	0.159363374
	chr1	89088241	89088372	132	0.150909091
	chr11	62376965	62377104	140	0.096112188
	chr21	27125	27260	136	0.06104778
	chr6	60658570	60658699	130	0.049090909
	chr6	42897341	42897470	130	0.022909091
	chr12	34623610	34623749	140	0.009979432
	chr1	11721	11850	130	0.002444444
	chr1	156190	156319	130	0.002444444
	chr1	170445	170574	130	0.002444444
	chr1	367066	367195	130	0.002444444
	chr1	13016105	13016234	130	0.002444444
	chr1	13212726	13212855	130	0.002444444

Table S2. GO terms.

	User ID	Gene Symbol	Gene Name	Entrez Gene
**HERV-K(HLM-2)** ***env***	ALG1L	ALG1L	ALG1, chitobiosyldiphosphodolichol beta-mannosyltransferase like	200810
	ATP23	ATP23	ATP23 metallopeptidase and ATP synthase assembly factor homolog	91419
	ATP4A	ATP4A	ATPase H+/K+ transporting subunit alpha	495
	BOC	BOC	BOC cell adhesion associated, oncogene regulated	91653
	C2CD4C	C2CD4C	C2 calcium dependent domain containing 4C	126567
	CCNYL1	CCNYL1	cyclin Y like 1	151195
	CD48	CD48	CD48 molecule	962
	CDH6	CDH6	cadherin 6	1004
	CEP126	CEP126	centrosomal protein 126	57562
	CEP97	CEP97	centrosomal protein 97	79598
	COL22A1	COL22A1	collagen type XXII alpha 1 chain	169044
	CR1	CR1	complement C3b/C4b receptor 1 (Knops blood group)	1378
	CR1L	CR1L	complement C3b/C4b receptor 1 like	1379
	DDX6	DDX6	DEAD-box helicase 6	1656
	DEFB107A	DEFB107A	defensin beta 107A	245910
	DEFB107B	DEFB107B	defensin beta 107B	503614
	DGCR2	DGCR2	DiGeorge syndrome critical region gene 2	9993
	DLGAP2	DLGAP2	DLG associated protein 2	9228
	FOXK1	FOXK1	forkhead box K1	221937
	KCNK9	KCNK9	potassium two pore domain channel subfamily K member 9	51305
	LHX8	LHX8	LIM homeobox 8	431707
	LIPH	LIPH	lipase H	200879

	LRIG3	LRIG3	leucine rich repeats and immunoglobulin like domains 3	121227
	MEI4	MEI4	meiotic double-stranded break formation protein 4	101928601
	METTL21A	METTL21A	methyltransferase like 21A	151194
	MSTO1	MSTO1	misato mitochondrial distribution and morphology regulator 1	55154
	NEPRO	NEPRO	nucleolus and neural progenitor protein	25871
	OR4F17	OR4F17	olfactory receptor family 4 subfamily F member 17	81099
	OR4F21	OR4F21	olfactory receptor family 4 subfamily F member 21	441308
	OSBPL11	OSBPL11	oxysterol binding protein like 11	114885
	PRKCQ	PRKCQ	protein kinase C theta	5588
	PRODH	PRODH	proline dehydrogenase 1	5625
	RPL24	RPL24	ribosomal protein L24	6152
	RPUSD3	RPUSD3	RNA pseudouridine synthase D3	285367
	SENP2	SENP2	SUMO specific peptidase 2	59343
	SFMBT2	SFMBT2	Scm like with four mbt domains 2	57713
	SGCD	SGCD	sarcoglycan delta	6444
	SLAMF1	SLAMF1	signaling lymphocytic activation molecule family member 1	6504
	SLC44A5	SLC44A5	solute carrier family 44 member 5	204962
	TDRP	TDRP	testis development related protein	157695
	THEG	THEG	theg spermatid protein	51298
	TMPRSS15	TMPRSS15	transmembrane serine protease 15	5651
	TREH	TREH	trehalase	11181
	TRPC6	TRPC6	transient receptor potential cation channel subfamily C member 6	7225
	YY1AP1	YY1AP1	YY1 associated protein 1	55249
	ZNF492	ZNF492	zinc finger protein 492	57615
	ZNF596	ZNF596	zinc finger protein 596	169270

	ZNF98	ZNF98	zinc finger protein 98	148198
**HERV-K(HLM-2) *gag***	AGTR1	AGTR1	angiotensin II receptor type 1	185
	ALG1L	ALG1L	ALG1, chitobiosyldiphosphodolichol beta-mannosyltransferase like	200810
	ATP23	ATP23	ATP23 metallopeptidase and ATP synthase assembly factor homolog	91419
	BOC	BOC	BOC cell adhesion associated, oncogene regulated	91653
	CDH6	CDH6	cadherin 6	1004
	CEP126	CEP126	centrosomal protein 126	57562
	COL22A1	COL22A1	collagen type XXII alpha 1 chain	169044
	DDX6	DDX6	DEAD-box helicase 6	1656
	DEFB107A	DEFB107A	defensin beta 107A	245910
	DEFB107B	DEFB107B	defensin beta 107B	503614
	DLGAP2	DLGAP2	DLG associated protein 2	9228
	FOXK1	FOXK1	forkhead box K1	221937
	KCNK9	KCNK9	potassium two pore domain channel subfamily K member 9	51305
	KMT2E	KMT2E	lysine methyltransferase 2E	55904
	LHFPL3	LHFPL3	LHFPL tetraspan subfamily member 3	375612
	LHX8	LHX8	LIM homeobox 8	431707
	LIPH	LIPH	lipase H	200879
	LRIG3	LRIG3	leucine rich repeats and immunoglobulin like domains 3	121227
	MEI4	MEI4	meiotic double-stranded break formation protein 4	101928601
	MSTO1	MSTO1	misato mitochondrial distribution and morphology regulator 1	55154
	NEPRO	NEPRO	nucleolus and neural progenitor protein	25871
	OR4F17	OR4F17	olfactory receptor family 4 subfamily F member 17	81099
	OR4F21	OR4F21	olfactory receptor family 4 subfamily F member 21	441308
	OSBPL11	OSBPL11	oxysterol binding protein like 11	114885

	PRODH	PRODH	proline dehydrogenase 1	5625
	SENP2	SENP2	SUMO specific peptidase 2	59343
	SGCD	SGCD	sarcoglycan delta	6444
	SLC44A5	SLC44A5	solute carrier family 44 member 5	204962
	TDRP	TDRP	testis development related protein	157695
	TMPRSS15	TMPRSS15	transmembrane serine protease 15	5651
	TREH	TREH	trehalase	11181
	TRPC6	TRPC6	transient receptor potential cation channel subfamily C member 6	7225
	YY1AP1	YY1AP1	YY1 associated protein 1	55249
	ZNF596	ZNF596	zinc finger protein 596	169270
**HERV-K(HLM-2)** ***pol***	ABCC2	ABCC2	ATP binding cassette subfamily C member 2	1244
	ALG10	ALG10	ALG10, alpha-1,2-glucosyltransferase	84920
	ALG1L	ALG1L	ALG1, chitobiosyldiphosphodolichol beta-mannosyltransferase like	200810
	ASRGL1	ASRGL1	asparaginase like 1	80150
	ATP23	ATP23	ATP23 metallopeptidase and ATP synthase assembly factor homolog	91419
	BOC	BOC	BOC cell adhesion associated, oncogene regulated	91653
	C6orf226	C6orf226	chromosome 6 open reading frame 226	441150
	CD48	CD48	CD48 molecule	962
	CDH6	CDH6	cadherin 6	1004
	CEP126	CEP126	centrosomal protein 126	57562
	CEP97	CEP97	centrosomal protein 97	79598
	DDX6	DDX6	DEAD-box helicase 6	1656
	DEFB107A	DEFB107A	defensin beta 107A	245910
	DEFB107B	DEFB107B	defensin beta 107B	503614
	DGCR2	DGCR2	DiGeorge syndrome critical region gene 2	9993

DLGAP2	DLGAP2	DLG associated protein 2	9228
DNMBP	DNMBP	dynamin binding protein	23268
ENSA	ENSA	endosulfine alpha	2029
FOXK1	FOXK1	forkhead box K1	221937
GBP1	GBP1	guanylate binding protein 1	2633
GBP2	GBP2	guanylate binding protein 2	2634
LIPH	LIPH	lipase H	200879
LRIG3	LRIG3	leucine rich repeats and immunoglobulin like domains 3	121227
MSTO1	MSTO1	misato mitochondrial distribution and morphology regulator 1	55154
NEPRO	NEPRO	nucleolus and neural progenitor protein	25871
OR4F17	OR4F17	olfactory receptor family 4 subfamily F member 17	81099
OR4F21	OR4F21	olfactory receptor family 4 subfamily F member 21	441308
OR4F29	OR4F29	olfactory receptor family 4 subfamily F member 29	729759
OR4F5	OR4F5	olfactory receptor family 4 subfamily F member 5	79501
OSBPL11	OSBPL11	oxysterol binding protein like 11	114885
PRAMEF13	PRAMEF13	PRAME family member 13	400736
PRAMEF18	PRAMEF18	PRAME family member 18	391003
PRAMEF27	PRAMEF27	PRAME family member 27	101929983
PRAMEF6	PRAMEF6	PRAME family member 6	440561
PRKCQ	PRKCQ	protein kinase C theta	5588
PRODH	PRODH	proline dehydrogenase 1	5625
PTCRA	PTCRA	pre T cell antigen receptor alpha	171558
RPL24	RPL24	ribosomal protein L24	6152
SCGB1A1	SCGB1A1	secretoglobin family 1A member 1	7356
SENP2	SENP2	SUMO specific peptidase 2	59343

	SFMBT2	SFMBT2	Scm like with four mbt domains 2	57713
	SGCD	SGCD	sarcoglycan delta	6444
	SLAMF1	SLAMF1	signaling lymphocytic activation molecule family member 1	6504
	TDRP	TDRP	testis development related protein	157695
	TMPRSS15	TMPRSS15	transmembrane serine protease 15	5651
	TREH	TREH	trehalase	11181
	TRPC6	TRPC6	transient receptor potential cation channel subfamily C member 6	7225
	YY1AP1	YY1AP1	YY1 associated protein 1	55249
	ZNF420	ZNF420	zinc finger protein 420	147923
	ZNF585A	ZNF585A	zinc finger protein 585A	199704
	ZNF596	ZNF596	zinc finger protein 596	169270

Table S3. Summary of pathways enriched in KEGG mapping.

GeneSet	Description	Size	Overlap	Expect	Enrichment Ratio	P Value	Overlap ID	Database	User ID
**HERV-K(HLM-2)** ***env***									
R-HSA-114508	Effects of PIP2 hydrolysis	7	2	0.063193481	31.64883402	0.001786048	5588;7225	pathway_Reactome	PRKCQ;TRPC6
R-HSA-1461957	Beta defensins	2	2	0.098300971	20.34567901	0.004287503	245910;503614	pathway_Reactome	DEFB107A;DEFB107B
R-HSA-373752	Netrin-1 signalling	0	2	0.117024965	17.09037037	0.006030302	5588;7225	pathway_Reactome	PRKCQ;TRPC6
R-HSA-1461973	Defensins	2	2	0.121705964	16.43304843	0.006508752	245910;503614	pathway_Reactome	DEFB107A;DEFB107B
R-HSA-3065679	SUMO is proteolytically processed	6	1	0.014042996	71.20987654	0.013964092	59343	pathway_Reactome	SENP2
R-HSA-5632681	Ligand-receptor interactions	8	1	0.018723994	53.40740741	0.018576919	91653	pathway_Reactome	BOC
R-HSA-6803157	Antimicrobial peptides	97	2	0.227028433	8.809469263	0.021426446	245910;503614	pathway_Reactome	DEFB107A;DEFB107B
R-HSA-3215018	Processing and activation of SUMO	10	1	0.023404993	42.72592593	0.023168962	59343	pathway_Reactome	SENP2
R-HSA-8877330	RUNX1 and FOXP3 control	10	1	0.023404993	42.72592593	0.023168962	1378	pathway_Reactome	CR1

	development of regulatory T lymphocytes (Tregs)								
R-HSA-418890	Role of second messengers in netrin-1 signalling	10	1	0.023404993	42.72592593	0.023168962	7225	pathway_Reactome	TRPC6
**HERV-K(HLM-2) *gag***									
R-HSA-1461957	Beta defensins	42	2	0.072815534	27.46666667	0.002358599	245910;503614	pathway_Reactome	DEFB107A;DEFB107B
R-HSA-1461973	Defensins	52	2	0.090152566	22.18461538	0.003594902	245910;503614	pathway_Reactome	DEFB107A;DEFB107B
R-HSA-3065679	SUMO is proteolytically processed	6	1	0.010402219	96.13333333	0.010359473	59343	pathway_Reactome	SENP2
hsa04925	Aldosterone synthesis and secretion	6	2	0.166435506	12.01666667	0.011811713	185;51305	pathway_KEGG	AGTR1;KCNK9
R-HSA-6803157	Antimicrobial peptides	7	2	0.168169209	11.89278351	0.012047913	245910;503614	pathway_Reactome	DEFB107A;DEFB107B
R-HSA-5632681	Ligand-receptor interactions	8	1	0.013869626	72.1	0.013789915	91653	pathway_Reactome	BOC
R-HSA-3215018	Processing and activation of	0	1	0.017337032	57.68	0.017209059	59343	pathway_Reactome	SENP2

	SUMO								
R-HSA-418890	Role of second messengers in netrin-1 signalling	0	1	0.017337032	57.68	0.017209059	7225	pathway_Reactome	TRPC6
R-HSA-189085	Digestion of dietary carbohydrates	11	1	0.019070735	52.43636364	0.018914405	11181	pathway_Reactome	TREH
R-HSA-1296346	Tandem pore domain potassium channels	2	1	0.020804438	48.06666667	0.02061694	51305	pathway_Reactome	KCNK9
**HERV-K(HLM-2) *pol***									
R-HSA-114508	Effects of PIP2 hydrolysis	7	2	0.072555479	27.5651135	0.002352528	5588;7225	pathway_Reactome	PRKCQ;TRPC6
R-HSA-1461957	Beta defensins	2	2	0.112864078	17.72043011	0.005627993	245910;503614	pathway_Reactome	DEFB107A;DEFB107B
R-HSA-6788656	Histidine, lysine, phenylalanine, tyrosine, proline and tryptophan catabolism	6	2	0.123613037	16.17952314	0.006720387	5625;80150	pathway_Reactome	ASRGL1;PRODH
R-HSA-373752	Netrin-1 signalling	0	2	0.134361997	14.88516129	0.007901241	5588;7225	pathway_Reactome	PRKCQ;TRPC6
R-HSA-1461973	Defensins	2	2	0.139736477	14.31265509	0.008524247	245910;503614	pathway_Reactome	DEFB107A;DEFB107B
R-HSA-3065679	SUMO is	6	1	0.01612344	62.02150538	0.016018957	59343	pathway_Reactome	SENP2

	proteolytically processed								
R-HSA-381753	Olfactory signalling pathway	93	4	1.056085298	3.787572847	0.020174897	441308;729759;79501;81099	pathway_Reactome	OR4F17;OR4F21;OR4F29;OR4F5
R-HSA-5632681	Ligand-receptor interactions	8	1	0.02149792	46.51612903	0.021303211	91653	pathway_Reactome	BOC
R-HSA-877300	Interferon gamma signalling	2	2	0.247226075	8.089761571	0.025180912	2633;2634	pathway_Reactome	GBP1;GBP2
R-HSA-3215018	Processing and activation of SUMO	0	1	0.026872399	37.21290323	0.026559998	59343	pathway_Reactome	SENP2

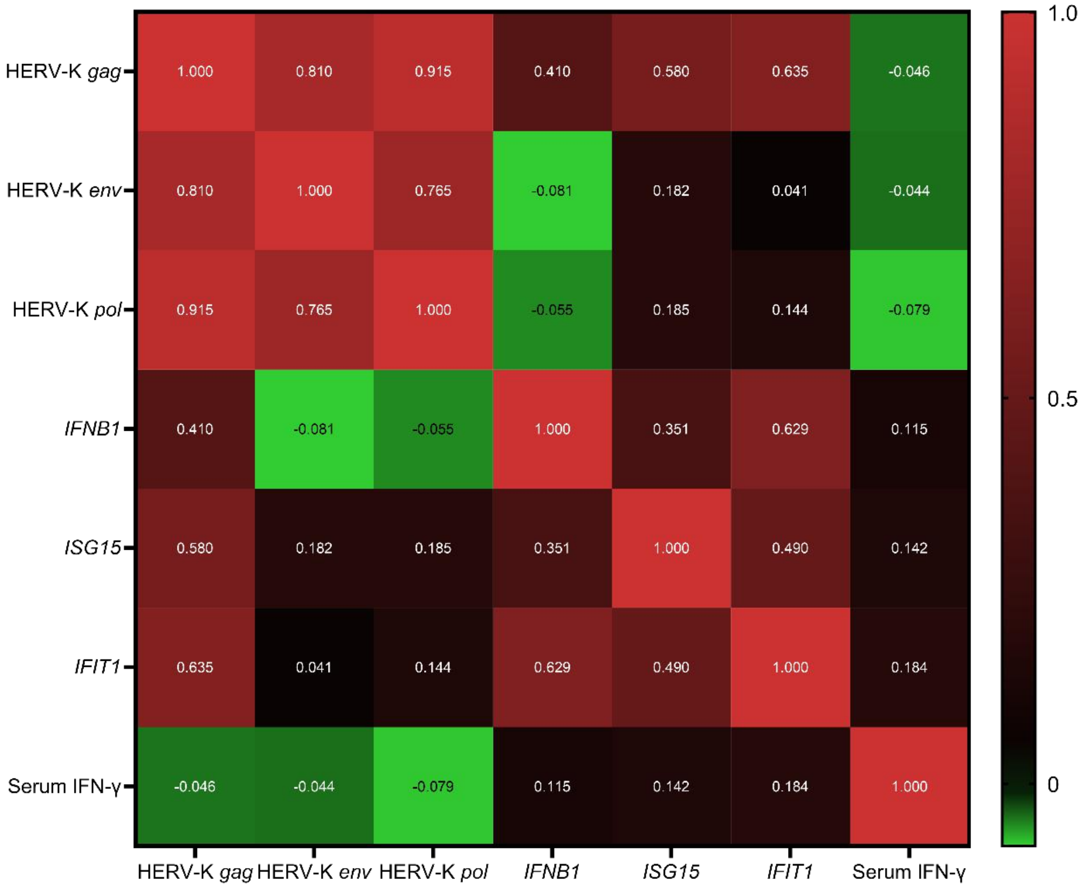

Figure S1. Correlation coefficient heat map.
